# Estimating Similarity of Dose–Response Relationships in Phase I Clinical Trials—Case Study in Bridging Data Package

**DOI:** 10.3390/ijerph18041639

**Published:** 2021-02-09

**Authors:** Adrien Ollier, Sarah Zohar, Satoshi Morita, Moreno Ursino

**Affiliations:** 1 INSERM, Centre de Recherche des Cordeliers, Sorbonne Université, USPC, Université de Paris, F-75006 Paris, France; adrien.ollier@inserm.fr (A.O.); moreno.ursino@inserm.fr (M.U.); 2 Department of Biomedical Statistics and Bioinformatics, Kyoto University Graduate School of Medicine, Kyoto 606-8501, Japan; smorita@kuhp.kyoto-u.ac.jp; 3 F-CRIN PARTNERS Platform, Assistance Publique-Hôpitaux de Paris, Université de Paris, F-75010 Paris, France

**Keywords:** bridging studies, distribution distance, oncology, phase I, dose-finding, dose–response, bayesian inference

## Abstract

Bridging studies are designed to fill the gap between two populations in terms of clinical trial data, such as toxicity, efficacy, comorbidities and doses. According to ICH-E5 guidelines, clinical data can be extrapolated from one region to another if dose–reponse curves are similar between two populations. For instance, in Japan, Phase I clinical trials are often repeated due to this physiological/metabolic paradigm: the maximum tolerated dose (MTD) for Japanese patients is assumed to be lower than that for Caucasian patients, but not necessarily for all molecules. Therefore, proposing a statistical tool evaluating the similarity between two populations dose–response curves is of most interest. The aim of our work is to propose several indicators to evaluate the distance and the similarity of dose–toxicity curves and MTD distributions at the end of some of the Phase I trials, conducted on two populations or regions. For this purpose, we extended and adapted the commensurability criterion, initially proposed by Ollier et al. (2019), in the setting of completed phase I clinical trials. We evaluated their performance using three synthetic sets, built as examples, and six case studies found in the literature. Visualization plots and guidelines on the way to interpret the results are proposed.

## 1. Introduction

Bridging studies are designed to fill the gap between two populations in terms of clinical trial data, such as toxicity, efficacy, comorbidities and doses. A bridging data package consists of selected data from the Clinical Data Package of the population in the new region, including pharmacokinetic, any pharmacodynamic, dose–toxicity or dose–efficacy data, and if appropriate, a bridging study to extrapolate the foreign dose–response data to the new region [1].

According to the International Council for Harmonisation of Technical Requirements for Pharmaceuticals for Human Use E5 (ICH-E5) guidelines, data can be extrapolated from one region to another if “a bridging study [...] indicates that a different dose in the new region results in a safety and efficacy profile that is not substantially different from the one derived from the original region; it will often be possible to extrapolate the foreign data to the new region, with an appropriate dose adjustment, if this can be adequately justified (e.g., by pharmacokinetic and/or pharmacodynamic data)” [1]. This is the reason why proposing a statistical tool evaluating the similarity between two foreign dose–response curves is of great interest. If this is proven, then, other clinical trials data can be used and extrapolated for the new region.

In Japan, the Pharmaceuticals and Medical Devices Agency (PMDA) recommends the re-evaluation of a drug if there are insufficient data from Japanese patients [2]. Indeed, Phase I clinical trials in oncology, which aim to estimate the maximum tolerated dose (MTD), are often repeated. Ogura et al. [3] pointed out that MTD differences between populations could be due to the different distribution of genetic polymorphisms in enzymes involved in drug metabolism or of biomarker incidences in different populations. In particular, in Japan, Phase I trials are repeated based on a physiological/metabolic paradigm: MTDs for Japanese patients are often lower than the ones of for Caucasian patients [4]. Based on this assumption, Maeda and Kurokawa [5] have performed an intensive study comparing the MTD of 21 molecularly targeted cancer drugs in Japanese versus Caucasian populations. They found out that this assumption does not hold well: in their study, the MTD was lower for Japanese patients in only two cases, there were no differences between the two populations with 10 drugs and MTD was incommensurable as the evaluated dose range acted different with nine drugs. Moreover, Mizugaki et al. [6] have analyzed data of single-agent Phase I trials at the National Cancer Center Hospital between 1995 and 2012, comparing the dose-limiting toxicity (DLT) profiles and MTDs of Japanese trials with the trials from Caucasian populations.

Recently, methods for bridging dose-finding design have been proposed where previous population data were used to either calibrate the prior distribution of the Bayesian model parameter(s) or to choose the “working model” of the design for prospective trials [7]. Liu et al. [8] proposed using a Bayesian model to average the dose-finding method where the previous trial data were used to build three different skeletons which would then be averaged during the study. Moreover, Takeda and Morita recently defined an “historical-to-current” parameter that could describe the degree of borrowing from one population to the other [9]. Ollier et al. [10] proposed a bridging method where a borrowing parameter was estimated sequentially in a response adaptive design which quantifies the amount of reasonable borrowing according to the similarity between the two populations’ estimates. Usually, the proposed methods focus on one parameter, strictly related to the MTD and not on the full dose–toxicity response curve. All these methods were proposed with the purpose of using the foreign data to plan and conduct the future Phase I trial in the new region. Indeed, at this stage, the idea is to use the foreign data to calibrate model-based priors to be used in the new region trial. However, in most cases, the trial in the new region will not be planned this way, but rather by using the MTD information from the foreign region only, if available. The sophisticated statistical approach will not be used.

Another option is to compare the two dose–response curves estimated from each region and to evaluate how similar they are. In this case, the overall purpose is different from before; if the curves prove to be similar (under the uncertainty estimation), the new purpose will be to extrapolate other trial data—such as that of Phase II—to the new region and to avoid further repetition of clinical investigations. For dose–response curves, Bretz et al. [11] introduced an asymptotic test to evaluate the difference of the *minimum efficient dose* among several groups of subjects, according to a threshold. However, this method was built for later clinical phases and presents weaknesses when applied to a small sample size. By contrast, Bayesian methods could mitigate the issue of estimation based on a small sample size setting, since they do not rely on asymptotic approximations and prior distributions can be used to ensure more stability in computation. Thereafter, the degree of similarity could be considered directly at the posterior distributions level. Therefore, methods proposing to estimate the similarity between dose–toxicity curves should be proposed when there is the need to evaluate if the safety data can be extrapolated or not.

The aim of our work is to propose some Bayesian indicators that evaluate the distance and the similarity of (1) dose–toxicity curves, taking into account the variability, (2) the MTD posterior distributions, by extending and adapting the commensurability criterion initially proposed by Ollier et al. [10]. These indicators were applied to several Phase I trials presented in Maeda and Kurokawa [5] and Mizugaki et al. [6], evaluating the similarity between Western dose–toxicity data to Eastern ones. The proposed tools should be used by trial stakeholders in order to decide if other trials data could be extrapolated from the new region, and, if so, to avoid the repetition of multiple clinical trials. In the next section, the original commensurability parameter is summarized along with the proposed extensions and the dose–toxicity model used. The case studies are described in Section 3, while Section 4 details the computational settings. The results are given in Section 5, followed by a Discussion section.

## 2. Methods

In this section, we briefly recall the Bayesian commensurability measure used in Ollier et al. [10], which was originally adopted into a power prior setting [12]; we then propose extensions and modifications to this measure to be applied at the end of the study. We also introduce the Bayesian dose–toxicity model, which will be used for retrospective data analyses.

Let $D_{c}$ denote the Caucasian data, $D_{c}={\left\{\left(y_{j},x_{j}\right)\right\}}_{n_{c}}$, $n_{c}$ the sample size of $D_{c}$, and $y_{j}$ the binary outcome of the *j*-th patient which received dose $x_{j}$. In a similar way, we can define $D_{a}$, the Japanese data and associated parameters. Let us also set a model for the probability of toxicity vs dose; $p_{T}\left(x\right)=f\left(x,\beta \right)$, where f(.) denotes a convenient monotonous link function parametrized by β. The likelihood function for each population can be written as $L\left(\beta |D_{m}\right)=\prod_{j=1}^{n_{m}}f{\left(x,\beta \right)}^{y_{j}}{\left(1-f(x,\beta )\right)}^{1-y_{j}},$ for m=c,a.

### 2.1. Commensurability Distances

Ollier et al. [10] suggested to consider the likelihood function as a distribution, divided by a normalization constant. This type of normalized likelihood can also be seen as the resulting Bayesian posterior distribution when constant (probably improper) priors are used for the analysis. Then, the authors defined a measure of “commensurability” between the two data-sets through a distance $d(D_{c},D_{a})$, the Hellinger one, in the parameters space via the following relation

(1) $$d^{2}\left(D_{c},D_{a}\right)=\frac{1}{2}\int {\left(\sqrt{\frac{L{\left(\beta |D_{c}\right)}^{\min\left(1,\frac{n_{a}}{n_{c}}\right)}}{\int L{\left(\beta |D_{c}\right)}^{\min\left(1,\frac{n_{a}}{n_{c}}\right)}\;d\beta }}-\sqrt{\frac{L{\left(\beta |D_{a}\right)}^{\min\left(1,\frac{n_{c}}{n_{a}}\right)}}{\int L{\left(\beta |D_{a}\right)}^{\min\left(1,\frac{n_{c}}{n_{a}}\right)}\;d\beta }}\right)}^{2}d\beta .$$

The commensurability measure, denoted by γ, is then defined as $\gamma =d^{q}\left(D_{c},D_{a}\right)$, with $q\in R^{+}$. Values of *q* higher than 1 will reduce the computed distance, while values lower than 1 will lead to a more conservative method, increasing the computed distance. In case of sequential trials, the authors proved that, when coupled with the power prior approach, a conservative value of γ leads to a better result in terms of operating characteristics, as a percentage of the right MTD selection. However, at the end of the trial, we are interested in comparing the achieved results, without any discount in the resulting distance. Therefore, in this paper, we will focus on the original Hellinger distance, which is q=1. This computed distance is a positive number between 0 and 1, it tends towards the maximum value when the two datasets are quite different, and towards zero when they are close to each other. Each likelihood is divided by a normalization constant in order to ensure that it can be viewed as a probability distribution. The variance of the likelihood density depends on the sample size of the trial. To make the two likelihoods comparable in terms of precision (variance), if $n_{c}>n_{a}$, $L(\beta |D_{c})$ is raised to a power of less than 1, otherwise, $L(\beta |D_{a})$ is raised to a power of less than 1. Following this method, the variance of likelihood density of the trial with more patients is increased to almost fit the one of the trial with fewer patients. Practical examples are given in Ollier et al. [10].

A straightforward modification of the distance in Equation (1) was performed by changing the underlying flat prior into a proper one. The posterior distribution obtained with the weighted likelihood is then used in the Hellinger formula. Thus, denoted by $\pi_{post,c}\left(\beta |D_{c}\right)\propto L{\left(\beta |D_{c}\right)}^{\min\left(1,\frac{n_{a}}{n_{c}}\right)}\pi_{prior}\left(\beta \right)$ and by $\pi_{post,a}\left(\beta |D_{a}\right)\propto L{\left(\beta |D_{a}\right)}^{\min\left(1,\frac{n_{c}}{n_{a}}\right)}\pi_{prior}\left(\beta \right)$ the posterior distribution of β given $D_{c}$ and $D_{a}$, respectively, we have

(2) $$d_{mod}^{2}\left(D_{c},D_{a}\right)=\frac{1}{2}\int {\left(\sqrt{\pi_{post,c}\left(\beta |D_{c}\right)}-\sqrt{\pi_{post,a}\left(\beta |D_{a}\right)}\right)}^{2}d\beta .$$

This modification will ensure more stability in computation when the likelihoods involve more than one parameter. When flat/constant priors are used for $\pi_{prior}\left(\beta \right)$, Equation (2) is equivalent to Equation (1). Even if, theoretically, two different priors can be chosen for the two trials, we suggest using a single one for the sake of comparability.

Both previous distances work at the parameter level. They check if the whole dose–toxicity curve is similar or not. Using a single parameter model for the dose–toxicity relationship, as a one parameter logistic model used in the continual reassessment method (CRM) [13], is also equivalent to check the MTD distance. However, in models with more parameters, such as the Bayesian Logistic Regression Model (BLRM) [14] where we have two parameters, intercept and slope, we check if the bivariate distribution of β is the same. Since the distance is difficult to interpret in case of the multidimensional parameters space, we propose a summary distance using the resulting posterior MTD distribution. In our setting, the MTD, $x^{*}$, is estimated as the dose linked to a pre-specified toxicity target τ, that is, $x^{*}=f^{-1}\left(\tau |\beta \right)$, where $f^{-1}\left(.\right)$ is the inverse function of f(.). The posterior MTD distribution, $\pi_{MTD,m}\left(x^{*}|D_{m}\right)$, is obtained evaluating $x^{*}$ through the posterior distribution of the parameter, $\pi_{post,m}\left(\beta |D_{m}\right)$, for m=c,a. Therefore, we can define

(3) $$d_{MTD}^{2}\left(D_{c},D_{a}\right)=\frac{1}{2}\int {\left(\sqrt{\pi_{MTD,c}\left(x^{*}|D_{c}\right)}-\sqrt{\pi_{MTD,a}\left(x^{*}|D_{a}\right)}\right)}^{2}dx^{*}.$$

Note that this distance always involves a one dimensional integral.

Previous distances focused on understanding the similarity of the whole dose–toxicity curve between two populations. However, even with different slopes and intercepts, two populations can still have the same MTD. Those differences should generally indicate a difference in responsiveness to a drug and it is important to know when MTDs are similar but not the underlying curves. Therefore, we propose to couple the distances, previously described, with a measure denoting the difference in MTD point estimations. We can build this measure as a percentage using the median of the posterior MTD distributions, such as

(4) $$d_{p1}\left(D_{c},D_{a}\right)={\left(\frac{{\mathrm{med}}_{c}}{{\mathrm{med}}_{a}}\right)}^{1-2I({\mathrm{med}}_{c}<{\mathrm{med}}_{a})}-1,$$

where I(.) is the indicator function, which assumes the value 1 if the statement in parentheses is true and zero otherwise, and $med_{i}$ with i=c,a, is the median of the posterior MTD distribution of Caucasians and Japanese, respectively. This formulation was chosen for its easy interpretation, indeed, we check how much the highest MTD differs in percentage in respect to the lowest one. For this reason, the formula implies the exponent $1-2I({\mathrm{med}}_{c}<{\mathrm{med}}_{a})$, which allows us to always have the highest estimate at the numerator, and the −1 term. Similarly to the three previous measures, Equation (4) tends to zero when the two MTDs are very similar. However, this measure does not have an upper bound. We propose the use of the median since it is less impacted by outliers than the mean. The *maximum a posteriori* is another possible candidate, that is

(5) $$d_{p2}\left(D_{c},D_{a}\right)={\left(\frac{{\tilde{x}}_{c}^{*}}{{\tilde{x}}_{a}^{*}}\right)}^{1-2I({\tilde{x}}_{c}^{*}<{\tilde{x}}_{a}^{*})}-1,$$

where

$${\tilde{x}}_{i}^{*}=\underset{x^{*}}{arg max}\pi_{MTD,i}\left(x^{*}|D_{i}\right).$$

To summarize, the first three measures *d*, $d_{mod}$, and $d_{MTD}$ are bounded between 0 and 1. Even if they are not built as percentages, their interpretation could be strictly linked to the percentage. Otherwise, the last two measures $d_{p1}$ and $d_{p2}$ have a ratio-like measure, lower bounded at 0. In practice, they give the information on the number of times the maximum MTD is higher than the lowest one.

### 2.2. Dose–Toxicity Model

In this section, we describe the model selected for the link function f(.). Instead of the CRM, originally used in Ollier et al. [10], which is better suited to prospective trials than retrospective analyses (retrospective CRM requires special techniques), we opted for a more flexible BLRM model, with two parameters, the intercept $\beta_{0}$ and the (logarithm of the) slope $\beta_{1}$ [14]. The dose–toxicity relationship is represented by

$$\mathrm{logit}\left\{p_{T}\left(x\right)\right\}=\beta_{0}+\exp\left(\beta_{1}\right)\log\left(\frac{x}{x_{r}}\right)$$

where $\beta \in R^{2}$, $x_{r}$ denotes a reference dose and $\exp(\beta_{1})$ assures a positive final slope in the model. In this case, $f^{-1}\left(.\right)$ is equal to the logit function and the BLRM formulation is similar to the one of Zheng and Hampson [15]. To close the Bayesian model, we suggest a bivariate normal distribution as prior for $\left(\beta_{0},\beta_{1}\right)$.

Following the described model, the final MTD is estimated as $x^{*}=x_{r}\exp\frac{\mathrm{logit}\left(\tau \right)-\beta_{0}}{\exp(\beta_{1})}$. In order to minimize the overdispersion generated by this formula, we compared the distribution of the log ratio of the MTD and the reference dose, $x^{**}=\log\left(x^{*}/x_{r}\right)$ (instead of the real MTD). Therefore, we have also changed Equations (4) and (5), accordingly, to the new formulation ($x^{**}$) in order to preserve the original distance meaning, that is $d_{p1}\left(D_{c},D_{a}\right)=\exp\left|{\mathrm{med}}_{c}-{\mathrm{med}}_{a}\right|-1$ and $d_{p2}\left(D_{c},D_{a}\right)=\exp\left|{\tilde{x}}_{c}^{*}-{\tilde{x}}_{a}^{*}\right|-1$.

Finally, in a previous sensitivity analysis (not shown), even when comparing the distribution of the log ratio of the MTD and the reference dose, we faced instability in computation due to the issue of outliers. We have found that truncating the posterior distribution of $x^{**}$ between the 10 and 90 percentiles gives a good compromise between preserving trial information and computation stability.

## 3. Case Studies

To show the results and the interpretation of the proposed measures, we first introduce four different synthetic datasets (1 for Caucasian and 3 for Japanese), to check the results when two datasets are similar or not. We fixed the Caucasian dataset first: setting τ equal to 0.3, the MTD at dose 600 mg/day. The same setting was used for the Japanese synthetic-1 set. Moreover, the two datasets were generated to have the same dose–toxicity shape. Japanese synthetic-2 set shares the same MTD with the Caucasian set, but has a different dose–toxicity shape: the Japanese dose–toxicity is steeper at the MTD than the Caucasian one. The Japanese synthetic-3 set has a different dose–toxicity curve and MTD (200 mg/day). The data are summarized in Table 1.

Then, we applied our methods to eight examples found in the literature. Our research started by looking at the drugs presented in Maeda and Kurokawa [5] and Mizugaki et al. [6]. We selected only drugs for which both Caucasian and Japanese trial data were available. We then extracted the number of toxicities and the number of allocated patients to the administered doses in each trial. All those data are shown in Table 2, each time with the reference article. The MTD declared at the end of the trial is shown in a box. As we can see from Table 2, Caucasians and Japanese trials were not usually used with the same set of doses.

## 4. Settings

We chose τ, the target toxicity probability, to be used to define the MTD, which equals 0.3 for the three synthetic set examples, while it equals 0.25 for the real case studies. Most of real case studies followed an algorithm base allocation; therefore, it seemed more natural to have a threshold lower than 0.3, which is more frequently used when model based designs are adopted in oncology.

A non-informative bivariate prior distribution, commonly used in this setting, was chosen for the BLRM model as follows:

$$\left(\begin{array}{c} \beta_{0}\\ \beta_{1} \end{array}\right)∼N\left(\left(\begin{array}{c} \mathrm{logit}(0.1)\\ \log1 \end{array}\right),\left[\begin{array}{cc} 4 & 0\\ 0 & 4 \end{array}\right]\right).$$

The hyperprior parameters of the bivariate prior were chosen after a preliminary sensitivity analysis (not shown) in order to ensure computational stability. In detail, this prior choice suggests a mean prior probability of toxicity at the reference dose, $x_{r}$, of 0.1 and that the slope has the prior median centered at zero. Therefore, $x_{r}$ was chosen in the first half of the total dose panel for each example. In detail, 400 mg/day was set for the three synthetic examples, 1 mg/m^2^ for Erilubin, 900 mg/day for Lapatinib, 200 mg/day for Sorafenib, 30 mg/m^2^ for Ixabepilone, 8 mg/m^2^ for Edotecarin and 700 mg/m^2^ for E7070.

All distances were computed with q=1, which is why we focus on the square root of Equation (1)–(3) and on the original value for Equation (4) and (5). The reference doses selected are reported along with the results in Table 3. All computations were performed in R, version 3.5.2. Monte Carlo approximations were adopted for all integrals involved, and uniform prior distribution on compact supports was set to approximate weighted likelihoods (as posterior distributions) in Equation (4). Details can be found in R scripts in the Supplementary Materials.

## 5. Results

The computed distances under all the proposed methods are shown in Table 3. When the MTD and the dose–toxicity curves are similar, like in synthetic-1 data, *d*, $d_{mod}$, $d_{MTD}$ are lower than 0.23 and $d_{p1}=d_{p2}=0$. When only the MTDs are similar (synthetic-2 data) but not the dose–toxicity curves, $d_{p1}=d_{p2}=0.02$ but *d*, $d_{mod}$, $d_{MTD}$ are higher than 0.37. Finally, when both curves and MTDs (synthetic-3 data) differ $d_{p1}=1.50$, $d_{p2}=1.27$ and *d*, $d_{mod}$, $d_{MTD}$ are higher than 0.83.

**Figure 1:**
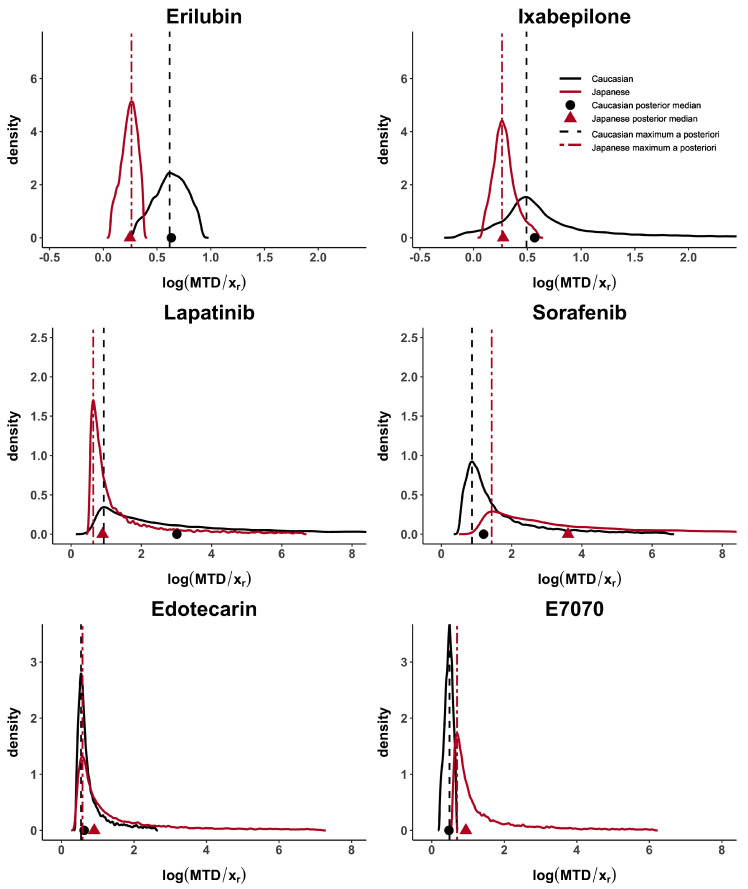
MTD posterior distributions for Erilubin, Ixabepilone, Lapatinib, Sorafenib, Edotecarin and E7070 case studies. Posterior medians are represented by a circle for Caucasian and a triangle for Japanese, while *maximum a posteriori* is represented by a dashed line for Caucasian and a two-dash line for Japanese.

Taking these cases’ studies as reference, we then analyse the data from published papers with Caucasian and Japanese datasets. Erilubin has the highest values of *d*, $d_{mod}$ and $d_{MTD}$, greater than 0.80, which suggests differences between the dose–toxicity curves. It is also shown in Figure 1. Its values of $d_{p1}$ and $d_{p2}$ are around 0.45. Ixabepilone and E7070 have quite large *d*, $d_{mod}$ and $d_{MTD}$, greater than 0.56 and they also have similar results in term of $d_{p2}$. The value of $d_{p1}$ is different in these two examples and reflects the presence of unbalanced heavy tails in the E7070 case. The heavy tail concern is observed, in at least one population, in all examples except for Erilubin. The results obtained in Table 3 show that $d_{p1}$ is directly impacted by this phenomenon. For example, Lapatinbib and Sorafenb have a very high value of $d_{p1}$, greater than 7.29, whereas the maximum a posteriori, $d_{p1}$, has more stable and usual results. Edotecarin has close values of *d*, $d_{mod}$ and $d_{MTD}$, around 0.3, representing similar dose–toxicity curves.

**Figure 2:**
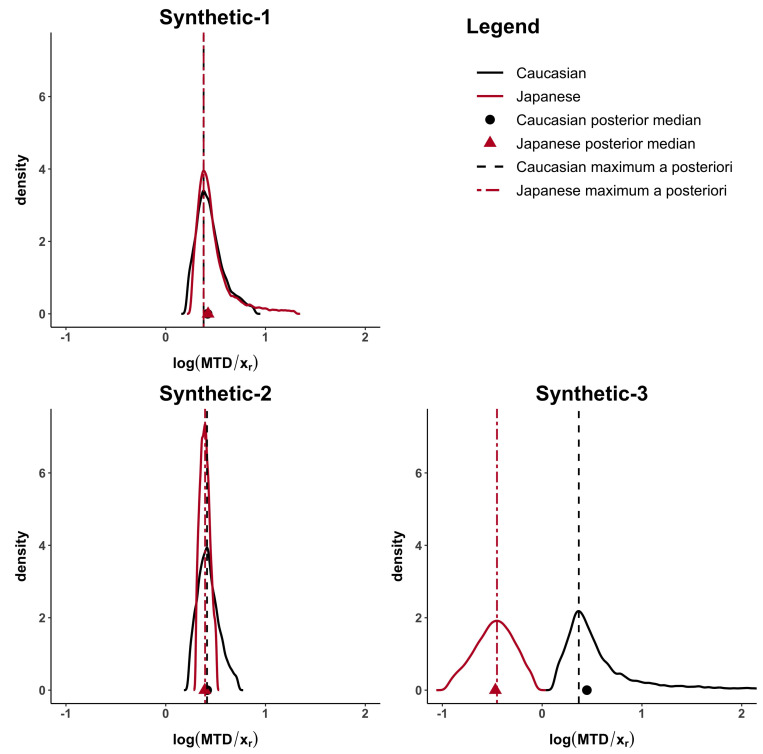
MTD posterior distributions for the Synthetic-1, Synthetic-2 and Synthetic-3 examples. Posterior medians are represented by a circle for Caucasian and a triangle for Japanese, while *maximum a posteriori* by a dashed line for Caucasian and a two-dash line for Japanese.

**Figure A1:**
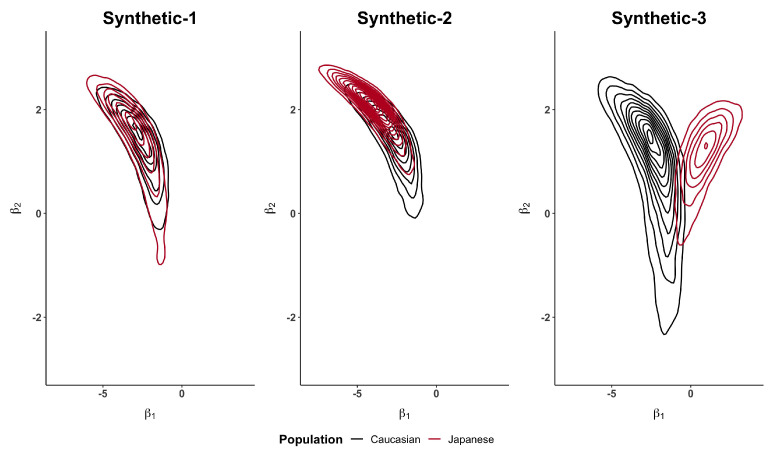
Bivariate posterior distributions of $\beta_{0}$ and $\beta_{1}$ when using $d_{mod}$ for the three synthetic examples.

Figure 2 and Figure A1, in the Appendix A, show how the Caucasian posterior distribution is different in the three synthetic examples even if it comes from the same Caucasian dataset. This behaviour is due to the variance adjustment given by $\min\left(1,\frac{n_{a}}{n_{c}}\right)$. In general, the posterior peak is preserved and the variance increases when the exponent is less than 1 (as in the synthetic-3 example).

**Figure 3:**
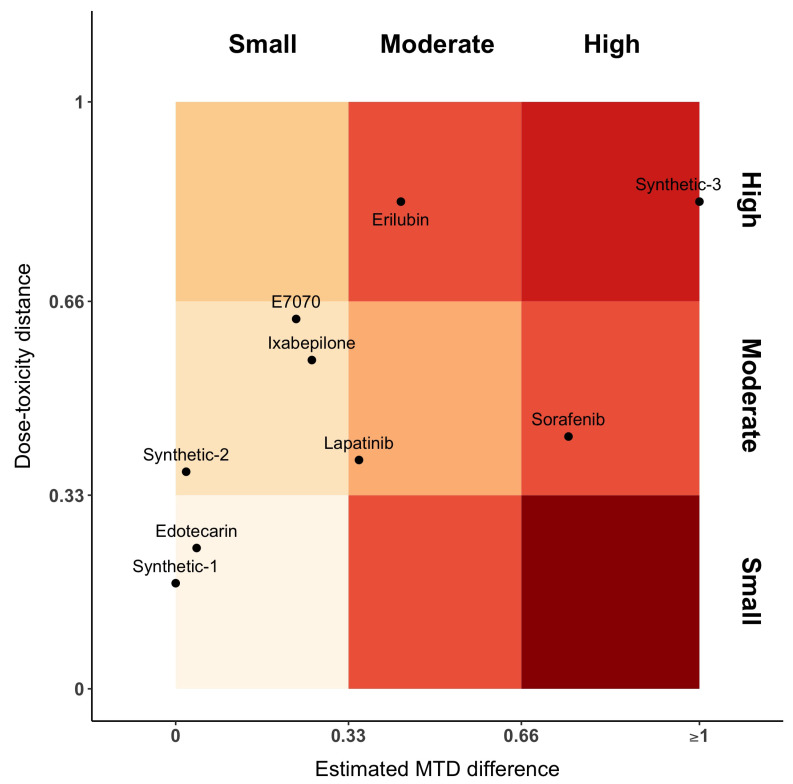
Gradient plot representing the distance between dose–toxicity curves, $d_{mod}$ (*y*-axis), and maximum of the posterior MTD distribution, $d_{p2}$ (*x*-axis). The intensity of the color varies along with the increasing distance value and coherence. Small dose–toxicity distance and high MTD distance is incoherent, as such it is plotted in a darker color.

Figure 3 represents the distance between dose–toxicity curves, $d_{mod}$, and maximum of the posterior MTD distribution, $d_{p2}$. For the sake of interpretability, we have equally divided the axes into three parts, each one denoting a small, moderate or high distance, respectively. In this plot, Sorafenib has moderate distances between curves and high difference between MTDs. This is the opposite for Erilubin, where there is a moderate difference between MTD and a large distance between curves. When MTDs are similar or close (first column of the gradient), Edotecarin has similar dose–toxicity curves, while the distance between curves of Ixabepilone and E7070 is moderate. Lapatinib shows a moderate distance of both dose–toxicity curve and estimated MTDs.

## 6. Discussion

The aim of our work was to propose several Bayesian indicators to support further decisions when using a bridging data package [1]. Bayesian methods permit the definition of a similarity degree based on posterior distribution, which do not rely on asymptotic approximations and can be used also in small sample size settings. Specifically, we proposed Bayesian indicators which evaluate the distance and the similarity of dose–toxicity curves and MTD. When evaluating a drug among different populations, assessing the dose–response curves similarity is of most importance, since, if it is proved, other clinical trial data can be used, as well as extrapolation from one population to the other. Maeda and Kurokawa [5] pointed out the difficulty of defining a commensurability measure for different populations.

We presented and studied five criteria, where three of them, *d*, $d_{mod}$ and $d_{MTD}$, measure the similarity between dose–toxicity curves, and two of them, $d_{p1}$ and $d_{p2}$, measure the distance between the median and the *maximum a posteriori* of the MTD posterior distributions. The first three measures are bounded between 0 and 1 and their interpretation could be linked to a proportion. The second ones, $d_{p1}$ and $d_{p2}$ have a ratio-like value with a lower bound at 0. In practice, they represent a relative risk measure.

Our approach allows for the identification and discussion of similarities and differences between dose–toxicity curves and MTDs. However, as small samples were used in these studies, estimation of the entire dose–toxicity curve, when only part of the doses in the panel were evaluated, is complex and leads to an estimation with high variability. This is reflected in the values of *d*, $d_{mod}$ and $d_{MTD}$, which in our real case studies were above 0.2. When high differences between *d* and $d_{mod}$ are observed, this is probably due to computational difficulties in Equation (1), especially in computing the weighted likelihood without a stabilization term. In general, $d_{mod}$ is lower than $d_{MTD}$. This could be expected for two reasons: (i) $d_{MTD}$ introduces, via the transformation, more variability (increased in the density estimation step); (ii) $d_{MTD}$ is computed after truncating the posterior induced distribution of the MTD. Moreover, we showed that $d_{p2}$, based on the maximum a posteriori, is more stable than $d_{p1}$, which is based on the median, in the presence of unbalanced heavy tails. Therefore, $d_{p2}$ could be suggested as a more reliable measure in this setting. We have attempted the analysis while varying the variance matrix of the bivariate normal prior distribution and $d_{p1}$ was less stable (results not shown).

The MTD definition can vary according to the trial and to the population. Therefore, even if the same MTD is claimed in both Caucasian and Japanese populations, our analysis can identify differences. For instance, in the Japanese trial of Sorafenib, 400 mg/day is defined in the clinical trial as the MTD, but at the closest higher dose level, 600 mg/day, only one patient experienced toxicities (16.7%). Otherwise, in the Caucasian trial, three patients out of seven experienced toxicity at 600 mg/day (42.6%). Even if the two trials find the same MTD, the toxicity probability associated with each one differs. That is the reason why our results showed otherwise. Indeed, in the published clinical trials, there is a discrepancy between the method section defining the MTD and the real given MTD at the end of the trial. Our methods are based on data only and allow for evaluation of the actual similarity.

We decided to present the plot of the posterior densities (of the parameters and of the MTD) as it shows the super-position (or not) of the information. Plotting directly one-dimensional dose–response curves could, instead, be misleading and give hazardous interpretation.

A first limitation of our work is that we used published data, where the reporting can be sometimes incomplete in terms of DLTs and doses. For instance, in the paper of Burris et al. [18], we had to re-compose the DLT table and the dose-allocation sequence. Therefore, some interpretation discrepancy can be found in our Table 2. The issue of poor reporting in cancer trials was already raised by Zohar et al. [28] and Comets and Zohar [29]. As a second limitation, we did not provide fixed cut-offs for each criterion. In our opinion, the choice of the cut-offs depends on the application and on the quantity of information in the two trials. The more information we have, the more stringent cut-offs can be considered. Figure 3 only represents a proposition on the way to display the results.

The criteria proposed in this manuscript may be extended to be used in other settings. For example, when several trials are available, a meta-analysis of the dose–toxicity curves or of the MTDs can be considered [30,31,32]. In this case, pairwise distances can be previously estimated, in an empirical Bayes approach, and then be used to model the heterogeneity parameter(s) or to set prior distribution(s). Other extensions, which do not involve necessarily Phase I studies, could be considered: (i) in adults–children extrapolation; (ii) when we are interested to jointly evaluate efficacy and toxicity [33]; (iii) when comparing outcomes (efficacy or toxicity) of the same drug in different indications; (iv) when dealing with similarities in subgroups; (v) in comparing historical control data with respect to the actual trial in randomized Phase III trials.

Being able to quantify distance and bridging between two populations at the end of early Phase I trials can be useful to better characterize the dose–toxicity relationship and differences. In case of small or acceptable differences, the extrapolation process can be considered, as suggested in the ICH-E5.

## Figures and Tables

**Table 1:** Number of dose-limiting toxicity and total number of patients accrued at each dose for 1 Caucasian trial and 3 Japanese synthetic trials. In the first column, the trial population is specified. A dash (-) means that the dose was not tested in the specified population. A box denotes the dose that has been defined as maximum tolerated dose (MTD).

	Doses					
**Example (mg/Day)**	**100**	**200**	**400**	**500**	**600**	**800**
Caucasian (DLTs/nb pt)	0/3	0/3	0/6	-	3/9	2/3
Japanese						
Synthetic-1 (DLTs/nb pt)	-	-	-	1/10	2/8	2/2
Synthetic-2 (DLTs/nb pt)	-	-	0/3	0/9	4/12	3/3
Synthetic-3 (DLTs/nb pt)	0/3	1/6	3/3	-	-	-

**Table 2:** Value of dose-limiting toxicity and total number of patients accrued at each dose for all trials analysed in this manuscript. In the first column, the trial population is specified. A dash (-) means that the dose was not tested in the specified population. A box denotes the dose that has been defined as MTD, if the MTD was reached in the trial. For Sorafenib, the doses were given twice daily (bid).

Investigated Drug	Doses								
**Erilubin (mg/m^2^)**	**0.25**	**0.5**	**0.7**	**1.0**	**1.4**	**2**	**2.8**	**4**	
Caucasian [16] (DLTs/nb pt)	0/1	0/4	-	0/3	-	1/7	2/3	3/3	
Japanese [17] (DLTs/nb pt)	-	-	0/3	0/3	2/6	3/3	-		
**Lapatinib (mg/day)**	**500**	**650**	**900**	**1000**	**1200**	**1600**	**1800**		
Caucasian [18] (DLTs/nb pt)	0/13	1/15	0/11	1/3	1/12	1/13	-		
Japanese [19] (DLTs/nb pt)	-	-	0/6	-	0/6	1/6	1/6		
**Sorafenib (mg bid)**	**100**	**200**	**400**	**600**					
Caucasian [20] (DLTs/nb pt)	0/3	1/6	0/8	3/7					
Japanese [21] (DLTs/nb pt)	0/3	1/12	0/6	1/6					
**Ixabepilone (mg/m^2^)**	**7.4**	**15**	**30**	**40**	**50**	**57**	**65**		
Caucasian [22] (DLTs/nb pt)	0/3	0/3	0/3	-	3/22	3/3	2/3		
Japanese [23] (DLTs/nb pt)	-	0/3	0/3	1/6	2/2	-	-		
**Edotecarin (mg/m^2^)**	**6**	**8**	**11**	**13**	**15**				
Caucasian [24] (DLTs/nb pt)	0/3	0/3	0/6	1/9	4/9			
Japanese [25] (DLTs/nb pt)	-	0/3	1/6	1/9	2/62/6				
**E7070 (mg/m^2^)**	**50**	**100**	**200**	**400**	**600**	**700**	**800**	**900**	**1000**
Caucasian [26] (DLTs/nb pt)	0/4	0/3	0/3	0/3	0/4	2/7	2/4	-	3/3
Japanese [27] (DLTs/nb pt)	-	-	-	0/3	0/3	0/6	1/6	2/3	-

**Table 3:** Results in terms of *d*, $d_{mod}$, $d_{MTD}$, $d_{p1}$ and $d_{p2}$ for the synthetic examples and the real case studies. $x_{r}$ denotes the reference dose selected for the Bayesian Logistic Regression Model (BLRM).

Drug	*d*	$d_{\mathrm{mod}}$	$d_{\mathrm{MTD}}$	$d_{p1}$	$d_{p2}$
Synthetic-1	0.23	0.18	0.19	0	0
Synthetic-2	0.53	0.37	0.41	0.02	0.02
Synthetic-3	0.91	0.83	1.00	1.50	1.27
Erilubin	0.92	0.83	0.91	0.47	0.43
Lapatinib	0.58	0.39	0.50	7.29	0.35
Sorafenib	0.45	0.43	0.57	10.07	0.75
Ixabepilone	0.77	0.56	0.62	0.34	0.26
Edotecarin	0.38	0.24	0.32	0.32	0.04
E7070	0.63	0.63	0.88	0.59	0.23

**Figure A2:**
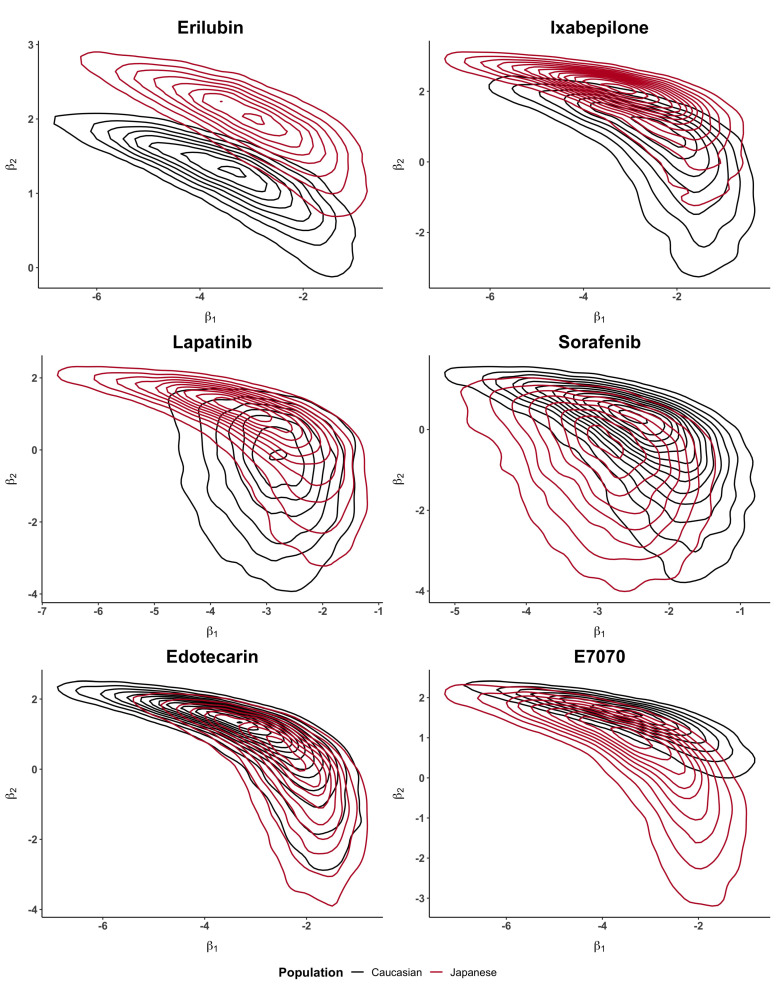
Bivariate posterior distributions of $\beta_{0}$ and $\beta_{1}$ when using $d_{mod}$ for the real case studies shown in Table 2.

## Data Availability

R scripts are given as Supplemental Materials.

## Supplementary Materials

The following are available at https://www.mdpi.com/1660-4601/18/4/1639/s1.

## Abbreviations

The following abbreviations are used in this manuscript:

bid	*bis in die*: twice a day
BLRM	Bayesian Logistic Regression Model
CRM	Continual reassessment method
DLT	dose-limiting toxicity
ICH	International Conference on Harmonisation of Technical Requirements for Registration of Pharmaceuticals for Human Use
MTD	maximum tolerated dose
PMDA	Pharmaceuticals and Medical Devices Agency

## Appendix A. Bivariate Posterior Plots

Figure A1 and Figure A2 show the bivariate posterior distributions of $\beta_{0}$ and $\beta_{1}$ when using $d_{mod}$.

## References

[1] ICH E5 (R1) (1998). Ethnic Factors in the Acceptability of Foreign Clinical Data E5 (R1).

[2] Pharmaceuticals and Medical Devices Agency (2015). Basic Principles for Conducting Phase I Trials in the Japanese Population Prior to Global Clinical Trials. https://www.pmda.go.jp/files/000157777.pdf.

[3] Ogura T., Morita S., Yonemori K., Nonaka T., Urano T. (2014). Exploring Ethnic Differences in Toxicity in Early-Phase Clinical Trials for Oncology Drugs. Ther. Innov. Regul. Sci..

[4] Malinowski H.J., Westelinck A., Sato J., Ong T. (2008). Same drug, different dosing: Differences in dosing for drugs approved in the United States, Europe, and Japan. J. Clin. Pharmacol..

[5] Maeda H., Kurokawa T. (2014). Differences in maximum tolerated doses and approval doses of molecularly targeted oncology drug between Japan and Western countries. Investig. New Drugs.

[6] Mizugaki H., Yamamoto N., Fujiwara Y., Nokihara H., Yamada Y., Tamura T. (2015). Current status of single-agent phase I trials in Japan: Toward globalization. J. Clin. Oncol..

[7] O’Quigley J., Iasonos A. (2014). Bridging Solutions in Dose Finding Problems. Stat. Biopharm. Res..

[8] Liu S., Pan H., Xia J., Huang Q., Yuan Y. (2015). Bridging continual reassessment method for phase I clinical trials in different ethnic populations. Stat. Med..

[9] Takeda K., Morita S. (2015). Incorporating Historical Data in Bayesian Phase I Trial Design: The Caucasian-to-Asian Toxicity Tolerability Problem. Ther. Innov. Regul. Sci..

[10] Ollier A., Morita S., Ursino M., Zohar S. (2020). An adaptive power prior for sequential clinical trials—Application to bridging studies. Stat. Methods Med. Res..

[11] Bretz F., Möllenhoff K., Dette H., Liu W., Trampisch M. (2016). Assessing the similarity of dose response and target doses in two non-overlapping subgroups. Stat. Med..

[12] Ibrahim J.G., Chen M.H., Gwon Y., Chen F. (2015). The power prior: Theory and applications. Stat. Med..

[13] O’Quigley J., Pepe M., Fisher L. (1990). Continual reassessment method: A practical design for phase 1 clinical trials in cancer. Biometrics.

[14] Neuenschwander B., Branson M., Gsponer T. (2008). Critical aspects of the Bayesian approach to phase I cancer trials. Stat. Med..

[15] Zheng H., Hampson L.V. (2020). A Bayesian decision-theoretic approach to incorporate preclinical information into phase I oncology trials. Biom. J..

[16] Tan A.R., Rubin E.H., Walton D.C., Shuster D.E., Wong Y.N., Fang F., Ashworth S., Rosen L.S. (2009). Phase I study of eribulin mesylate administered once every 21 days in patients with advanced solid tumors. Clin. Cancer Res. Off. J. Am. Assoc. Cancer Res..

[17] Mukohara T., Nagai S., Mukai H., Namiki M., Minami H. (2011). Eribulin mesylate in patients with refractory cancers: A Phase I study. Investig. New Drugs.

[18] Burris H.A., Hurwitz H.I., Dees E.C., Dowlati A., Blackwell K.L., O’Neil B., Marcom P.K., Ellis M.J., Overmoyer B., Jones S.F. (2005). Phase I Safety, Pharmacokinetics, and Clinical Activity Study of Lapatinib (GW572016), a Reversible Dual Inhibitor of Epidermal Growth Factor Receptor Tyrosine Kinases, in Heavily Pretreated Patients With Metastatic Carcinomas. J. Clin. Oncol. Off. J. Am. Soc. Clin. Oncol..

[19] Nakagawa K., Minami H., Kanezaki M., Mukaiyama A., Minamide Y., Uejima H., Kurata T., Nogami T., Kawada K., Mukai H. (2009). Phase I Dose-escalation and Pharmacokinetic Trial of Lapatinib (GW572016), a Selective Oral Dual Inhibitor of ErbB-1 and -2 Tyrosine Kinases, in Japanese Patients with Solid Tumors. Jpn. J. Clin. Oncol..

[20] Moore M., Hirte H., Siu L., Oza A., Hotte S., Petrenciuc O., Cihon F., Lathia C., Schwartz B. (2005). Phase I study to determine the safety and pharmacokinetics of the novel Raf kinase and VEGFR inhibitor BAY 43-9006, administered for 28 days on/7 days off in patients with advanced, refractory solid tumors. Ann. Oncol..

[21] Minami H., Kawada K., Ebi H., Kitagawa K., Kim Y.i., Araki K., Mukai H., Tahara M., Nakajima H., Nakajima K. (2008). Phase I and pharmacokinetic study of sorafenib, an oral multikinase inhibitor, in Japanese patients with advanced refractory solid tumors. Cancer Sci..

[22] Aghajanian C., Burris H.A., Jones S., Spriggs D.R., Cohen M.B., Peck R., Sabbatini P., Hensley M.L., Greco F.A., Dupont J. (2007). Phase I Study of the Novel Epothilone Analog Ixabepilone (BMS-247550) in Patients with Advanced Solid Tumors and Lymphomas. J. Clin. Oncol..

[23] Shimizu T., Yamamoto N., Yamada Y., Fujisaka Y., Yamada K., Fujiwara Y., Takayama K., Tokudome T., Klimovsky J., Tamura T. (2008). Phase I clinical and pharmacokinetic study of 3-weekly, 3-h infusion of ixabepilone (BMS-247550), an epothilone B analog, in Japanese patients with refractory solid tumors. Cancer Chemother. Pharmacol..

[24] Hurwitz H.I., Cohen R.B., McGovren J.P., Hirawat S., Petros W.P., Natsumeda Y., Yoshinari T. (2007). A phase I study of the safety and pharmacokinetics of edotecarin (J-107088), a novel topoisomerase I inhibitor, in patients with advanced solid tumors. Cancer Chemother. Pharmacol..

[25] Yamada Y., Tamura T., Yamamoto N., Shimoyama T., Ueda Y., Murakami H., Kusaba H., Kamiya Y., Saka H., Tanigawara Y. (2006). Phase I and pharmacokinetic study of edotecarin, a novel topoisomerase I inhibitor, administered once every 3 weeks in patients with solid tumors. Cancer Chemother. Pharmacol..

[26] Raymond E., ten Bokkel Huinink W., Taïeb J., Beijnen J., Faivre S., Wanders J., Ravic M., Fumoleau P., Armand J., Schellens J. (2002). Phase I and Pharmacokinetic Study of E7070, a Novel Chloroindolyl Sulfonamide Cell-Cycle Inhibitor, Administered as a One-Hour Infusion Every Three Weeks in Patients with Advanced Cancer. J. Clin. Oncol..

[27] Yamada Y., Yamamoto N., Shimoyama T., Horiike A., Fujisaka Y., Takayama K., Sakamoto T., Nishioka Y., Yasuda S., Tamura T. (2005). Phase I pharmacokinetic and pharmacogenomic study of E7070 administered once every 21 days. Cancer Sci..

[28] Zohar S., Lian Q., Levy V., Cheung K., Ivanova A., Chevret S. (2008). Quality assessment of phase I dose-finding cancer trials: Proposal of a checklist. Clin. Trials.

[29] Comets E., Zohar S. (2009). A survey of the way pharmacokinetics are reported in published phase I clinical trials, with an emphasis on oncology. Clin. Pharmacokinet..

[30] Zohar S., Katsahian S., O’Quigley J. (2011). An approach to meta-analysis of dose-finding studies. Stat. Med..

[31] Ursino M., Röver C., Zohar S., Friede T. (2019). Random-effects meta-analysis of phase I dose-finding studies using stochastic process priors. arXiv.

[32] Röver C., Friede T. (2019). Dynamically borrowing strength from another study through shrinkage estimation. Stat. Methods Med. Res..

[33] Thall P.F., Cook J.D. (2004). Dose-finding based on efficacy–toxicity trade-offs. Biometrics.

