# Supplementary material for: Estimating Similarity of Dose–Response Relationships in Phase I Clinical Trials—Case Study in Bridging Data Package
Source: Int J Environ Res Public Health. 2021 Feb 9;18(4):1639. doi: 10.3390/ijerph18041639 (PMC7916097; doi:10.3390/ijerph18041639)
Supplement: Supplementary file 1 [file ijerph-18-01639-s001.zip › submitted/README.rtf]

READMEThe scripts in this folder were used to obtain the results shown in the manuscript.Session information:R version 3.5.2 (2018-12-20)Platform: x86_64-apple-darwin15.6.0 (64-bit)Running under: macOS High Sierra 10.13.6attached base packages:[1] stats     graphics  grDevices utils     datasets  methods   base     other attached packages:[1] ggpubr_0.4.0       gridExtra_2.3      ks_1.11.5          rstan_2.19.2       ggplot2_3.1.1      StanHeaders_2.19.0To obtain the results of the paper:1) Run the “functions_distances.R” file to build the function necessary to the analyses; pay attention to have all files in the same folder, above all the .stan files.2) Run “synthetic_data.R” to obtain the results regarding the three synthetic data-sets. Numerical results are shown in the R console. 3) Run “res_examples.R” to obtain the results regarding the real example data-sets. Numerical results are shown in the R console. 
